# Polarization modulated spectroscopic ellipsometry-based surface plasmon resonance biosensor for *E. coli* K12 detection

**DOI:** 10.1038/s41598-024-78535-8

**Published:** 2024-11-07

**Authors:** Soraya Zangenehzadeh, Emil Agocs, Fenja Schröder, Nassima Amroun, Rebekka Biedendieck, Dieter Jahn, Axel Günther, Lei Zheng, Bernhard Roth, Hans-Hermann Johannes, Wolfgang Kowalsky

**Affiliations:** 1 Institut für Hochfrequenztechnik, Technische Universität Braunschweig, Brunswick, 38106 Germany; 2 Cluster of Excellence PhoenixD, Hannover, 30167 Germany; 3 Institute of Microbiology and Braunschweig Integrated Centre of Systems Biology, Technische Universität Braunschweig, Brunswick, 38106 Germany; 4 Hannover Centre for Optical Technologies, Leibniz University Hannover, Hannover, 30167 Germany

**Keywords:** Spectroscopic ellipsometry, Surface plasmon resonance, Electro-optic polarization modulation, *E. coli* bacteria, Optical sensors, Biophotonics

## Abstract

In this work, we report on the application of the polarization modulated spectroscopic ellipsometry-based surface plasmon resonance method for sensitive detection of microorganisms in Kretschmann configuration. So far, rotating analyzer and single wavelength polarization modulation methods have widely been investigated for phase sensitive surface plasmon resonance measurement. In this study, a much simpler optical setup relying on fast electro-optic phase modulator crystals is introduced for bacteria detection. A beta barium borate crystal connected to a function generator is adapted for generating phase shifts in the millisecond regime to extract the ellipsometric angles ($\Psi$ and $\Delta$) under the surface plasmon resonance condition. For detection, the gold surface was functionalized with anti-*Escherichia coli* antibodies, and *E. coli* K12 was attached to them. We show that polarization modulated spectroscopic ellipsometry achieves a refractive index resolution in the order of $10^{-5}$ RIU, and a limit of detection of $10^{2}$ CFU/mL for *E. coli* K12 which is compatible with other surface plasmon resonance based phase sensitive methods with more complex detection concepts. As a follow-up step, an optical model can be developed to enhance this biosensor’s performance, and applications for sorting and detecting other biological targets will be investigated.

## Introduction

Bacteria are a diverse group of single-celled microorganisms found almost everywhere on earth. The Gram-negative bacterium *Escherichia coli*, is a normal part of the intestinal flora of humans and animals^1,2^. It is a rod-shape bacterium with the size of approximately 1-3 $\mu$m. While most strains of *E. coli* are harmless, some can threaten human health dramatically by causing illnesses like urinary tract infection and kidney failure^1–4^. Every year, about 130 - 175 million cases of urinary tract infections occur word wide, and 75$\%$ are caused by *E. coli*^5^. Therefore, it is essential to detect pathogenic organisms at an early stage in e.g. drinking water or food. Enzyme-linked immunosorbent assay (ELISA) and polymerase chain reaction (PCR) are commonly used to detect such pathogenic microorganisms^6–10^. While these methods take advantage of high specificity and efficiency, their application is restricted to a limited number of specific known targets and long measurement time^11,12^. In contrast, optical biosensors with high sensitivity and accuracy can provide potential alternatives compared to traditional biosensing methods.

Optical biosensors are transducers that use light beams to convert biological or chemical interactions on their surfaces into measurable and interpretable signals^13,14^. Non-invasive and contactless analysis makes optical biosensors suitable for various applications, including drug delivery, microRNA analysis, food safety, and detection of disease caused by viruses and bacteria^15,16^. In the last two decades, surface plasmon resonance (SPR) based optical sensors have gained significant attention in the field of biosensing research due to their real-time and label-free measurements^17–19^. Surface plasmons (SPs) are collective oscillations of free electrons at a metal-dielectric interface where the sign of the real part of the dielectric function changes across the interface. These evanescent electromagnetic waves propagate at the metal-dielectric interface and penetrate exponentially into both media^20,21^. Different configurations like prism coupling and metallic gratings are introduced to transfer the energy of the incident photons to SPs and excite them^22^. The Kretschmann configuration, which relies on the attenuated total reflection (ATR) method, has a broad range of applications for biosensing^23–25^. This configuration consists of a high refractive index (RI) prism, a thin gold layer, and a dielectric medium. The mechanism behind the excitation of SPs involves coupling between the electromagnetic field of the incident light and the free electrons at the metal surface. The orientation of p-polarized light allows for strong coupling between the incident light and the SPs, leading to efficient excitation, where the reflectivity is significantly suppressed^18,22^. Optical properties (refractive index) of the ambient medium in contact with metallic films directly influence the dispersion relation of SPs. The changes in RI can be observed and accurately measured by optical techniques, including angular modulation and wavelength modulation^18,26–28^. In conventional SPR based biosensors, the normalized amplitude characteristics of reflected light are measured according to the angle of incidence (AOI), while other parameters are kept constant. In addition to measuring the reflectance, the phase change of the reflected light is strongly modulated under the resonance condition. Different methods are proposed to measure the phase changes such as optical heterodyne detection and ellipsometry^28–30^. While heterodyne-based phase measurement is fast and suitable for *in-situ* biological analysis, it is limited for spectroscopic measurement and also requires a complicated optical setup^31–33^. The ellipsometry technique suggests a non-destructive and non-contact optical setup which is mostly used to determine the optical properties of thin films. SPR-based ellipsometric biosensors can take advantage of more accurate detection when the contrast of the SPR resonance is poor due to low absorption losses of the dielectric stack that is sustaining the surface wave, which is not the case of SPP sustained by Au layer^34^. An ellipsometric approach to SPR-based sensors was first reported by Homola et al., where the implementation of polarization control advanced the detection of the SPR dip^35^. This technique determines the azimuth angle of elliptically polarized light by analyzing the measured intensities with known phase shifts^36–41^.

Spectroscopic ellipsometry (SE), measures the changes in the polarization state of the light beam after reflection from the target surface. SE provides information about the amplitude ratio and phase difference between s-and p-polarized states of light^42,43^. Ellipsometric angles are defined by a single complex quantity as follows:

1 $$\begin{aligned} \rho \equiv \tan {\Psi }\exp \left( {i\Delta }\right) \end{aligned}$$

Here, $\rho$ represents the complex reflectance ratio, $\Psi$ and $\Delta$ are the amplitude ratio and the phase difference between p-and s-polarizations, respectively. In general, two main experimental configurations for ellipsometric measurements are applied in SPR-based sensors. The first category involves mechanically rotating optical elements like rotating-analyzers^28,44^. Although this type of measurement takes advantage of simple optical configuration, measurement time increases due to the rotation frequency of 100 Hz^45^. The second category introduces SE measurements based on the modulation of the polarization state, and allows for high-resolution real-time studies^46–51^.

**Fig. 1:**
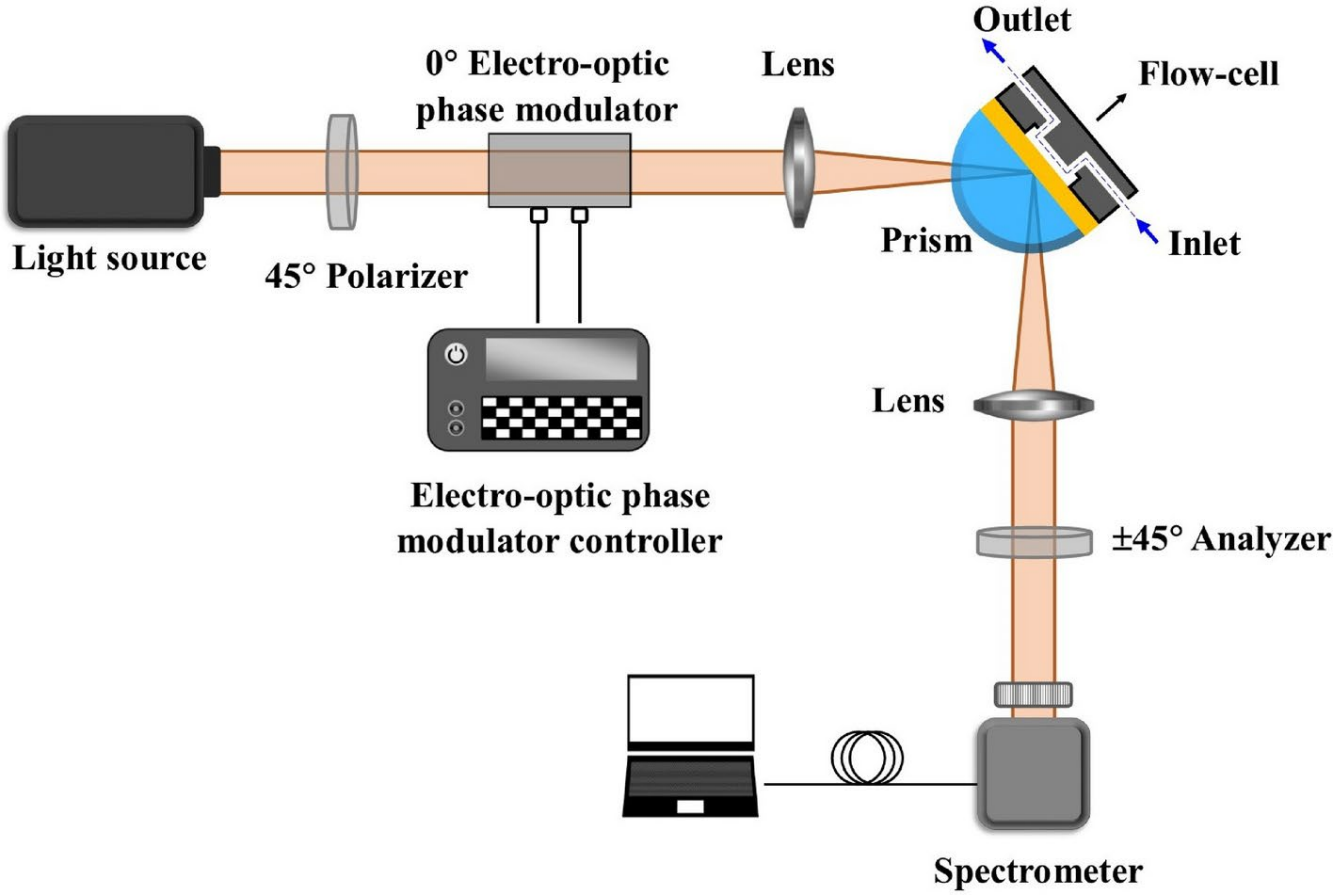
Schematic configuration of the optical setup based on polarization modulated spectroscopic ellipsometry using an electro-optic phase modulator.

In this work, we use a polarization modulated spectroscopic ellipsometer (PMSE) for the detection of *E. coli* K12 bacteria. Here, a SPR-based phase sensitive technique that relies on the spectroscopic ellipsometry measurement was used for the first time for monitoring *E. coli* bacteria. We find that the detection limit of the developed optical biosensor is competitive with conventional SPR-based biosensors^23–25^. At the same time, the method provides information about both phase and intensity modulation in a wide wavelength range with high time resolution. Figure 1 shows the schematic of the optical setup. To enhance the performance of the measurement, essential parameters including gold thickness and AOI were optimized using different water-glycerol concentrations. The optimization process was done using a commercial spectroscopic ellipsometer (SENpro, SENTECH Instruments GmbH, Berlin, Germany). Afterwards, the selected optimal parameters were applied in the developed PMSE for the detection process. The performance of the developed sensor including its resolution and sensitivity was assessed before the bacteria measurement. The notable feature of this PMSE is its ability for measuring the ellipsometric angles in the visible wavelength range (350 nm - 850 nm) with high time resolution on the order of milliseconds.

## Materials and methods

### Gold substrates preparation

The quartz glass wafers were purchased from Präzisions Glas & Optik GmbH (Iserlohn, Germany) with 0.7 mm thickness and were cut into pieces with a size of 25 mm x 25 mm. Substrates were washed with acetone and isopropanol in an ultrasonic bath for 30 minutes at room temperature. Finally, the substrates were dried with $\hbox {N}_{2}$. The physical vapor deposition method (combination of HHV Auto 306 and 500, HHV Ltd, West Sussex, United Kingdom) was used for gold deposition on substrates. First a 3 nm chromium thin film (at 0.2 (Å/s) evaporation rate) was formed on glass followed by the gold film with the desired thicknesses of 45 nm, 55 nm, and 60 nm (at 0.99 (Å/s) evaporation rate).

### Amine coupling method for antibody immobilization

**Fig. 2:**
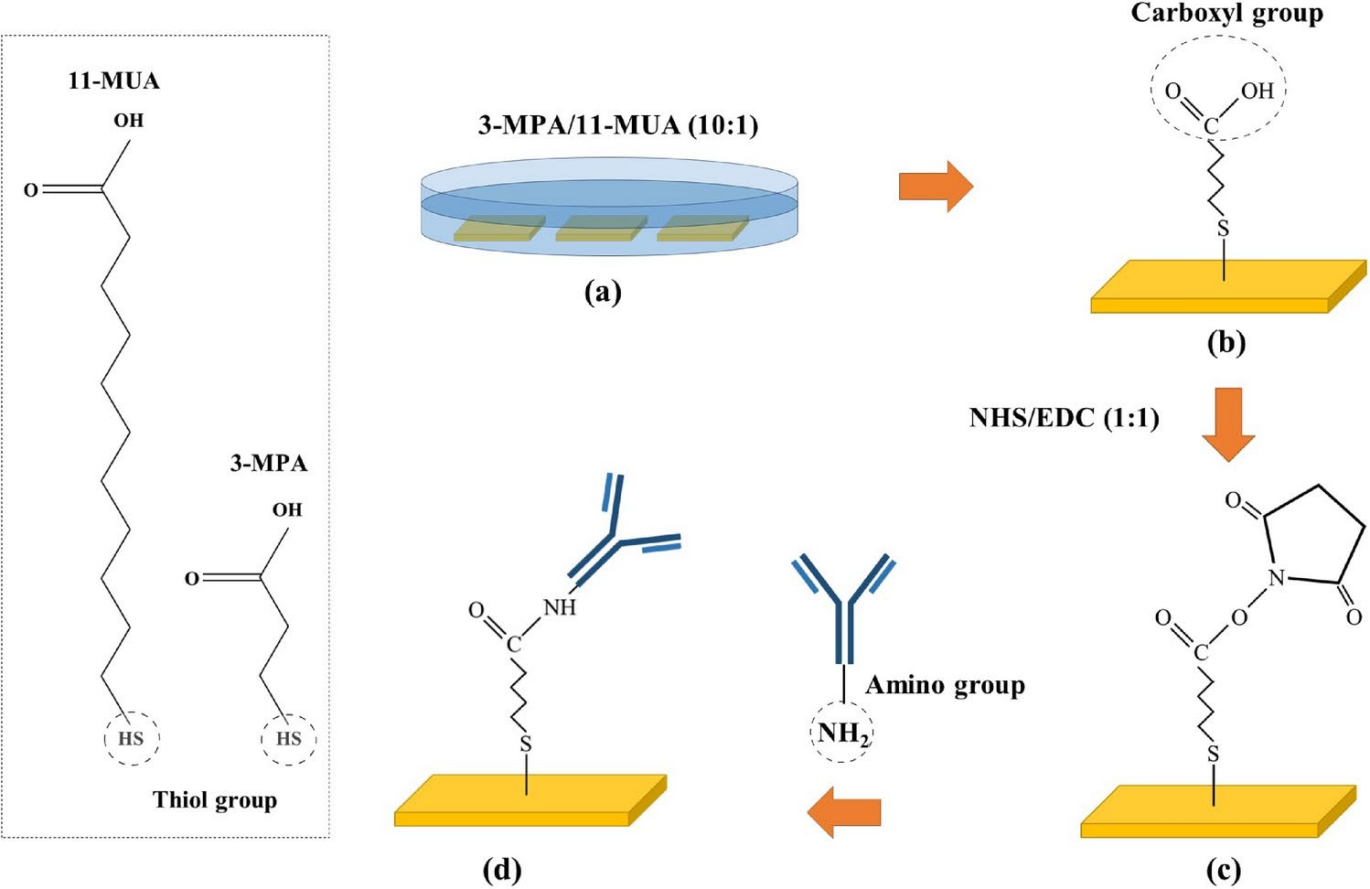
Schematic of the ligand immobilization process on the gold surface: (**a**) Self-assembled monolayer formation of carboxymethylated dextran chains on the gold surface, (**b**) Covalently bonded thiol group to the gold surface, (**c**) Sensor surface activation using EDC/NHS mixture and (**d**) Covalent bonding between anti-*E. coli* antibody and NHS-esters.

Chemicals were purchased from Sigma-Aldrich (Darmstadt, Germany). The immobilization process was started by self-assembled monolayer formation of a carboxymethylated dextran matrix on the gold surface. For this purpose the gold substrate was soaked in a 10:1 ratio mixture of 10 mM 3-MPA (3-Mercaptopropionic acid) and 10 mM 11-MUA (11-Mercaptoundecanoic acid) in ethanolic medium for 24 hours. In this reaction, the thiol group covalently binds to the gold to form Au-HS bonds^52–54^. Then the surface was washed with ethanol and deionized water (DI water) respectively and dried with $\hbox {N}_{2}$ gas. Then, the gold sample was positioned into the flow-cell. The chemicals were pumped with the speed of 6 $\mu$L/min onto the sensor surface. To activate the carboxyl groups of the self-assembled monolayer layer on the gold surface, a 1:1 mixture of 0.4 M EDC (*N*-(3-Dimethylaminopropyl)-*N*${'}$-ethyl-carbodiimid) and 0.1 M NHS (*N*- hydroxysuccinimide) was injected for 30 minutes. As a result of this activation, NHS-esters are produced which can covalently bind with ligands containing primary amino groups (–$\hbox {NH}_{2}$). In the next step, polyclonal rabbit anti-*E.* ${coli}$ antibody (Abcam Limited, Cambridge, United Kingdom) with 50 $\mu$g/mL concentration was inserted for 25 minutes. Finally, the surface was washed with 1M ethanolamine hydrochloride (Thermo Fisher Scientific, Bremen, Germany) for 10 minutes to deactivate the unreacted NHS-esters on the surface. The surface was washed with phosphate buffered saline (PBS, 0.14 mol/L NaCl, 2.7 mmol/L KCl, and 10 mmol/L phosphate, pH 7.4) as a running buffer between each chemical injections. The schematic of the immobilization process has been shown in Fig. 2.

### Bacteria culture and colony forming unit

The preculture was prepared by inoculating 50 mL of lysogeny-broth medium (LB, 10 g/L tryptone, 5 g/L yeast extract, 5 g/L sodium chloride) with cells from single colony of *E. coli* K12. The culture was incubated overnight at 37 $^ {\circ }$C with a rotation velocity of 150 rpm (Ecotron, Infors AG, Bottmingen, Switzerland). To prepare the main culture, preculture was diluted in LB medium with a 1:20 ratio, and incubated for around 4 hours until an optical density between 0.1 and 0.6 at a wavelength of 600 nm was reached (WPA biowave CO8000, Biochrom Ltd., Cambridge, United Kingdom). In the final step, a series of different bacteria concentrations were prepared by adding 1 mL of main culture to 9 mL of PBS. This dilution process was continued for six different orders of magnitude. To estimate the number of bacterial cells, 50 $\mu$L of each sample was poured on an agar plate and incubated overnight. Finally, the number of colonies was visually counted from the agar plate.

### Optical setup

The schematic configuration of the PMSE-based SPR biosensor is shown in Fig. 1. This figure illustrates the orientation of the optical elements for ellipsometric angle measurement, while for other purpose these parameters can be changed. For example, for SPR measurements, the polarizer and analyzer are set on $0 ^{\circ }$ to propagate p-polarized light. A broad spectrum light source (EQ-99X LDLS, Energetiq Technology, Inc, Wilmington, USA) was used in the ellipsometric optical setup. The light beam is directed to a wire grid polarizer (WP25M-VIS, Thorlabs GmbH, Bergkirchen, Germany) oriented at $45 ^{\circ }$, and then passes through the electro-optic phase modulator (PC3B-VIS, QUBIG GmbH, Munich, Germany) oriented at $0 ^{\circ }$ as well. The electro-optic phase modulator consist of a beta barium borate crystal which is connected to the function generator (from 0 V to +3200 V) and generates phase shifts with high time resolution on the order of milliseconds. After reflection from the gold-prism interface, the light beam propagates through the analyzer (second polarizer) with $\pm 45 ^{\circ }$ rotation angle and finally to the spectrometer (Ocean FX-XR1-ES, Ocean Optics, Maybachstrasse, Germany). Two lenses (f = 40 mm, LINOS®, Excelitas Technologies®  Corp., Waltham, USA) were placed before and after the flow-cell to focus the beam spot (700 $\mu$m) onto the measurement area.

## Results and discussion

The thickness of the thin gold film and the AOI have a significant impact on the performance of the SPR-based biosensors and excitation conditions. Here, three different gold thicknesses (45 nm, 55 nm, and 60 nm) and AOIs ($70 ^{\circ }$, $75 ^{\circ }$ and $80^{\circ }$) were examined, respectively to select the optimal thickness and angle. For this purpose, three different aspects were considered: I. the comparison of $\Psi$ and $\Delta$ spectra for the reference medium (pure water), II. the conventional sensitivity analysis for ellipsometric angles, and III. the sensitivity comparison between $\Psi$ and $\Delta$ spectra using the numerical integration analysis method.

**Fig. 3:**
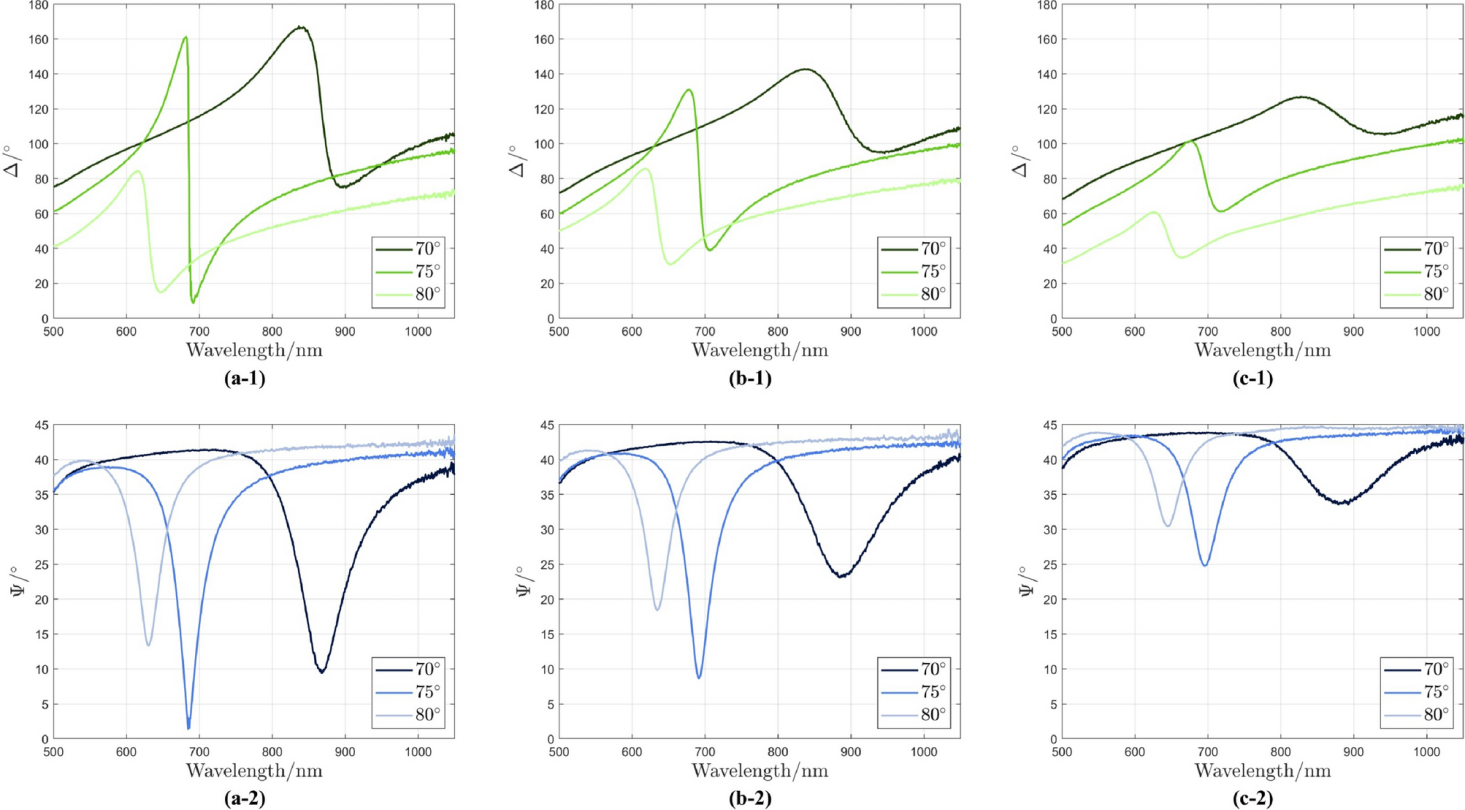
Ellipsometric angles ($\Psi$ and $\Delta$) for water as a reference medium for different gold film thicknesses and angles of incidence: (**a-1**) $\Delta$ for 45 nm, (**a-2**) $\Psi$ for 45 nm, (**b-1**) $\Delta$ for 55 nm, (**b-2**) $\Psi$ for 55 nm, (**c-1**) $\Delta$ for 60 nm, (**c-2**) $\Psi$ for 60 nm.

The commercial spectroscopic ellipsometer was used to measure the ellipsometric angles for pure water as the dielectric medium in contact with gold layer. A home-made Kretschmann flow-cell was designed using a half cylinder quartz prism (TECNOTTICA CONSONNI SRL, Calco LC, Italy) attached to the gold substrate with immersion oil. A peristaltic pump (LG-G100-1J-EU/DG-4-B, DARWIN microfluidics, Paris, France) controlled the speed of the water (70 $\mu$L/min). The measurement results are shown in Fig. 3. The left, middle, and right columns represent the $\Psi$ and $\Delta$ for 45 nm, 55 nm, and 60 nm, respectively. The sharp drops in $\Psi$ and $\Delta$ spectra indicates the SPR phenomena. As can be seen for the $\Psi$ spectra, the absorption decreases and FWHM increases with increasing gold thickness, which makes the signal less sensitive for further measurements. The resonance wavelength shifts from lower wavelengths ($\sim$ 640 nm) for $80^{\circ }$ AOI to higher wavelengths ($\sim$ 870 nm) for $70^{\circ }$ AOI. At $75^{\circ }$ AOI, the resonance wavelength is located close to 690 nm and shows the highest absorption and phase shift. As the gold layer thickness increases, the phase shift decreases for the $\Delta$ spectra (phase difference). For example, at $75^{\circ }$ AOI, the phase is shifted from $161.34^{\circ }$ to $8.79^{\circ }$ for a thickness of 45 nm at the resonance condition, while this parameter decreases for a thickness of 60 nm from $101.37^{\circ }$ to $61.16^{\circ }$. All three thickness values show lower phase shift at $70^{\circ }$ and $80^{\circ }$ compared to $75^{\circ }$. Overall, a 45 nm thin gold film at an AOI of $75^{\circ }$ represents the optimal result (highest absorption and phase shift).

Due to the broad obtained SPR spectra at $70^{\circ }$, the sensor’s performance was analyzed for $75^{\circ }$ and $80^{\circ }$ AOIs for each thickness. Sensitivity evaluation ($\Delta$ $\lambda _{SPR}$/$\Delta$n; $\lambda _{SPR}$ indicates the wavelength corresponding to the resonance condition) was performed using different water-glycerol concentrations (pure water, water-glycerol (1.25$\%$, 2.5$\%$, 5$\%$, 7.5$\%$, 10$\%$ and 12.5$\%$)) with different refractive indices. Here, the minimum of the resonance dip in the $\Psi$ spectrum indicates the $\lambda _{SPR}$ for $\Psi$, and the wavelength corresponding to the peak on the left-hand side in the $\Delta$ spectrum is considered as $\lambda _{SPR}$ for $\Delta$. The refractive index of the solutions was measured using an Abbe refractometer (64150, Carl Zeiss AG, Jena, Germany). Ellipsometric angles were measured starting from lowest (pure water) to highest (water-glycerol $12.5 \%$) concentrations. The maximum sensitivity is reported in Table 1 for both $\Psi$ and $\Delta$ at $75^{\circ }$ and $80^{\circ }$ AOIs for 45 nm, 55 nm, and 60 nm, respectively.

**Table 1:** Reported sensitivity for $\Psi$ and $\Delta$ in nm $\cdot$ $\hbox {RIU}^{-1}$.

		45 nm	55 nm	60 nm
$\Psi$	$75^{\circ }$	3304	3271	2751
	$80^{\circ }$	1624	1595	1565
$\Delta$	$75^{\circ }$	2956	3180	2436
	$80^{\circ }$	1683	1592	1488

Table 1 shows that the sensitivity at an angle of $80^{\circ }$ is lower compared to $75^{\circ }$ for all thicknesses. Also, with the AOI of $75^{\circ }$, highest sensitivity was achieved when 45 nm gold film was used. As the RI of the mixture changes, the resonance wavelength for $\Psi$ shifts, and the spectrum becomes broader or narrower. Consequently, the peak-to-valley distance in the $\Delta$ spectrum can also change. By tracking only the peaks and valleys in conventional sensitivity analysis, different values will be obtained. For this reason, to have a comprehensive analysis of the sensor’s performance for different thicknesses and AOIs, the numerical integration analysis method was also used. An analytical analysis was performed based on the difference of measured $\Psi$ and $\Delta$ spectra with respect to their spectra in the case of water, which is taken as the reference. The area under the curve (AUC) was calculated for each variation and divided by the signal error to compare the sensitivities of $\Psi$ and $\Delta$ in the same scale range (AUC[$\Psi _{n}$-$\Psi _{water}$]/error and AUC[$\Delta _{n}$-$\Delta _{water}$]/error, higher value means better sensitivity). The error is obtained by calculating the difference of two identical $\Psi$ and $\Delta$ signal for pure water as a reference.

**Fig. 4:**
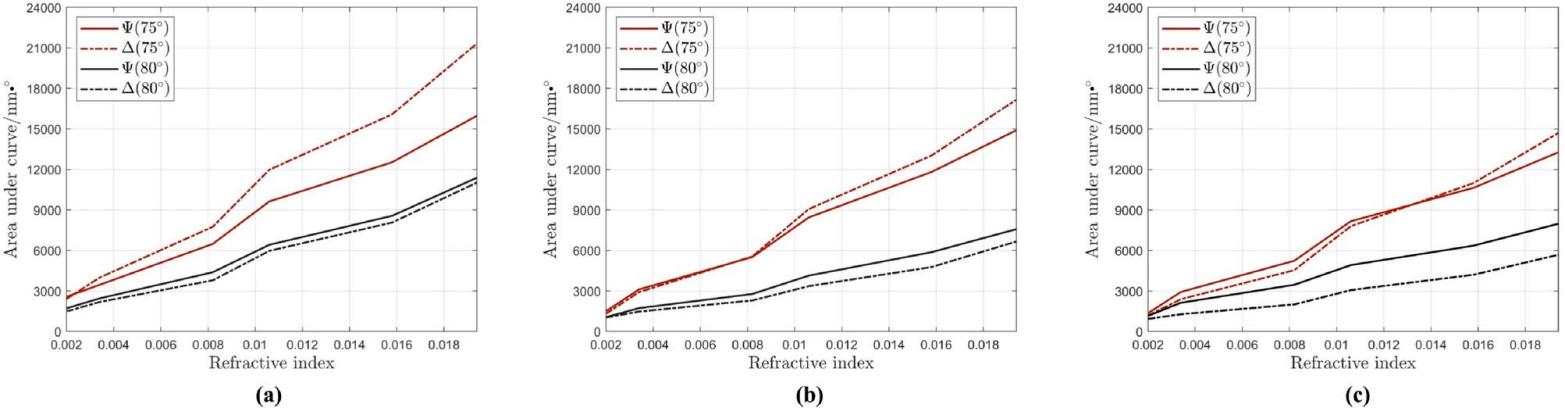
Calculated [area under the curve/error] for (**a**) 45 nm, (**b**) 55 nm and (**c**) 60 nm gold layer thicknesses versus the RI for different water-glycerol concentrations. Red and black curves present the data for $75^{\circ }$ and $80^{\circ }$, respectively. Solid and dashed lines represent the data for $\Psi$ and $\Delta$, respectively.

The results are shown in Fig. 4. Here, red and black curves show the results for $75^{\circ }$ and $80^{\circ }$, while the solid and dashed lines present $\Psi$ and $\Delta$ spectra, respectively. As shown in Fig. 4, at $75^{\circ }$ for 45 nm gold thickness, the $\Delta$ spectra are more sensitive to the changes of the RI than the $\Psi$ spectra. For lower RI changes, these two gradients are closer to each other, while they move away from each other when RI changes are larger. For the same thickness at $80^{\circ }$, $\Psi$ shows higher sensitivity, and both angles have the same gradient. By increasing the thickness to 55 nm, $\Psi$ and $\Delta$ show approximately the same sensitivity for RI changes below 0.008, and $\Delta$ is more sensitive for higher RI changes. The same study is repeated for 60 nm layer thickness, where the $\Psi$ spectra show higher sensitivity for RI changes below 0.013 while for the $\Delta$ spectra this is the case for higher RI changes. At $80^{\circ }$ for all three thicknesses, $\Psi$ is more sensitive than $\Delta$, and by increasing the thickness, this difference becomes more significant. By comparing all the data from the calibration part, a 45 nm thick gold sample at $75^{\circ }$ AOI was selected as optimal values for bacteria detection using the developed PMES-based SPR biosensor.

**Fig. 5:**
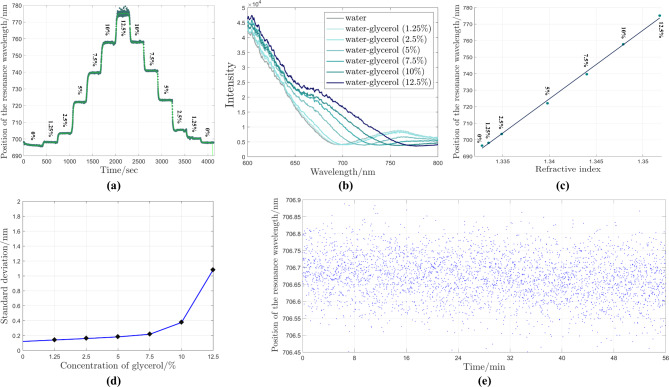
(**a**) *In-situ* measurement of the position of the resonance wavelength for different water-glycerol concentrations. Dark green and bright green curves represent the actual and smoothed data, respectively. (**b**) Intensity spectra near the plasmonic resonance wavelength for different water-glycerol concentrations. (**c**) Position of the resonance wavelength versus refractive index of water-glycerol solutions and fitted curve. (**d**) Calculated standard deviation for each water-glycerol concentration. (**e**) *In-situ* tracking of the position of the resonance wavelength for water as a reference medium for approximately 1 hour.

Prior to bacteria measurements, the performance of the developed PMSE was evaluated using the selected optimal parameters (45 nm thick gold film and $75^{\circ }$ AOI). In this configuration, the compensator has not been taken into account and both polarizer and analyzer were set to transmit p-polarized light. The sensitivity range of this configuration was assessed by bringing seven media with different refractive indices into contact with the gold film. Figure 5a shows the *in-situ* position of the resonance wavelength when the concentration of glycerol increases and subsequently decreases step by step. In *in-situ* measurement data, the resonance wavelength refers to the minimum intensity of the reflected light, which corresponds to the resonance wavelength. In Fig. 5b, as the concentration of glycerol increases, the resonance spectra become broader and the position of resonance wavelength is not detectable with high accuracy. While the sensor shows linear sensitivity in the range of 1.3327 RIU - 1.3521 RIU, signal fluctuations increase for higher concentrations (Fig. 5c). To estimate this fluctuation, the standard deviation (SD) for each concentration was calculated. As can be seen in Fig. 5d, the standard deviation increases linearly and slowly from 1.3327 (pure gold) to 1.3440 (7.5$\%$). The steeper slopes were seen for glycerol concentration of 10$\%$ and 12.5$\%$, which correlates with the broadened intensity spectra. Therefore, the best performance of this setup was found in the range of 1.3327 RIU - 1.3440 RIU with the sensitivity of 3,858 nm $\cdot$ $\hbox {RIU}^{-1}$, although solutions with higher RI can also be detected. Noise levels originating from the detection system influence the performance of the SPR-based sensors. Therefore, the evaluation of this parameter in the output spectra provides a valuable assessment of the stability of the sensor. The actual wavelength resolution of the detector is 0.4 nm. In order to calculate the full instrument resolution including the noise level and mechanical stability, the resonance wavelength fluctuations were measured for pure gold samples in DI water for 60 min with 1 second time step. The resonance wavelength was determined by fitting a third order polynomial curve close to the measured SPR region. The resonance wavelength fluctuation is shown in Fig. 5e and the calculated SD is 0.064 nm. Considering the SD calculation, the RI resolution of this developed PMSE is calculated (SD$\times$ $\Delta$n/$\Delta$ $\lambda _{SPR}$) to be 1.6$\times 10^{-5}$ RIU.

In SPR-based phase measurements, there are two main approaches which are utilized: angular modulation and wavelength modulation. Also, the data obtained from each classification are dependent on the polarimetric optical setup used, such as the rotating analyzer and the modulation of polarization. For angular modulation method, the measured refractive index resolution for both rotating analyzer and polarization modulation is between 2$\times 10^{-6}$ RIU - 6$\times 10^{-7}$ RIU^28,44,46–51^. For the wavelength modulation method, the RI resolution for the rotating analyzer method is in the order of $10^{-5}$ RIU^55^. Here, the ellipsometric angles were measured using the same method (wavelength modulation) but with polarization modulation. The refractive index resolution for this study is comparable with that of reference^55^, while the measurement speed is much faster because it is not limited to manually rotating elements. Although PMSE-based SPR biosensor has comparable RI resolution performance to laser-based SPR sensors, wavelength modulation offers several advantages over angular modulation (laser-based SPR sensors). Biomolecular interactions can be detected with wavelength modulated SPR sensors due to their wide dynamic detection range. Wavelength modulation enables real-time monitoring of the reflected light intensity and wavelength. With this capability, data can be acquired and analyzed faster, which is particularly beneficial for high-throughput applications. For instance, the designed PMSE-based biosensor here can achieve results in the order of milliseconds, making it suitable for rapid diagnostics and real-time monitoring applications.

**Fig. 6:**
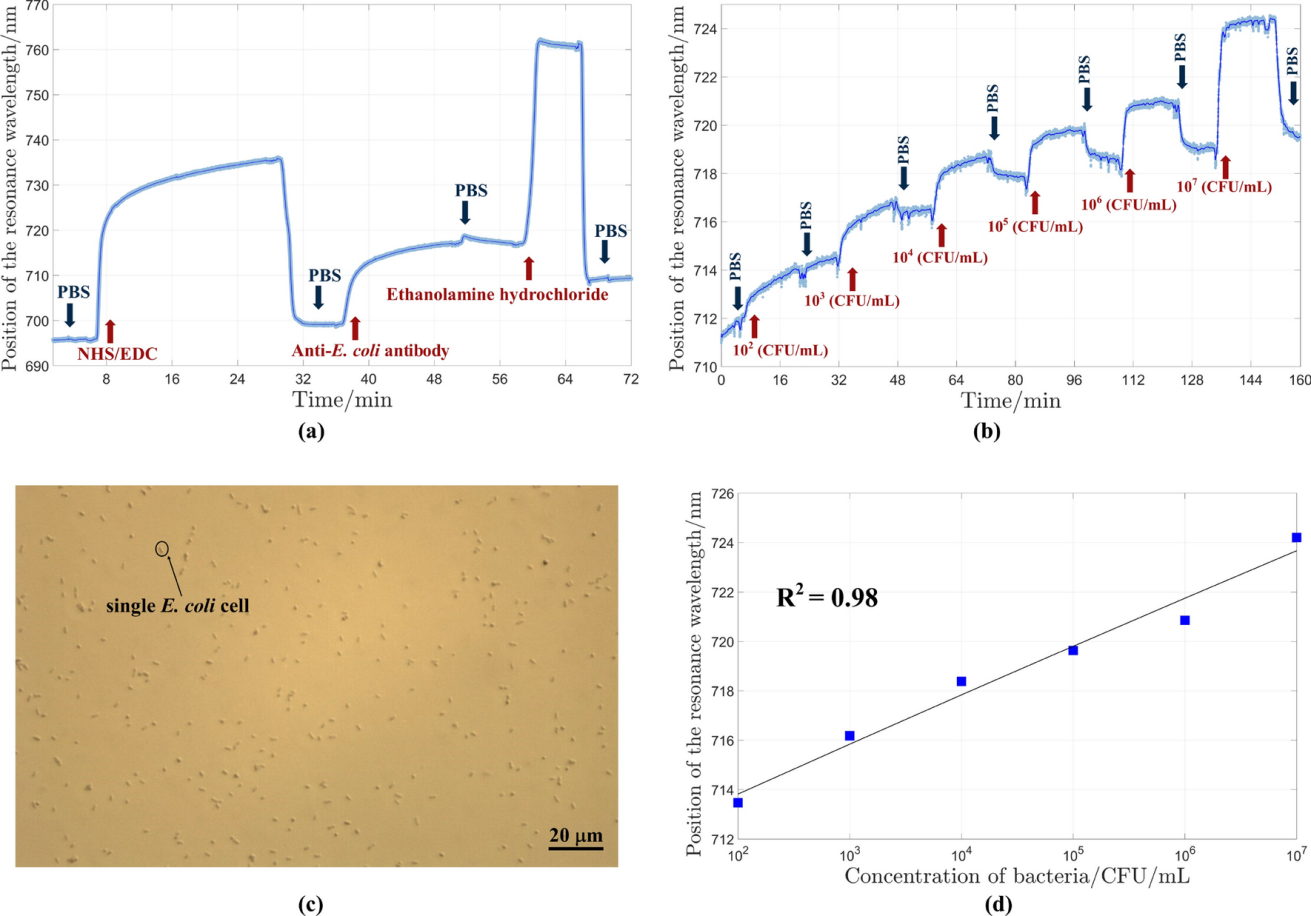
(**a**) Sensogram of ligand immobilization steps on sensor surface using the amine coupling method. (**b**) Sensogram of bacteria injections through the surface from lowest to highest concentrations. Between each injection, PBS was used to wash the surface. (**c**) Optical image of attached single *E. coli* K12 cell on gold surface after the final washing step with PBS. (**d**) The position of the resonance wavelength for each concentration of bacteria and calculated quadratic fit with high correlation of $\hbox {R}^2$ = 0.98.

Binding of target bacteria with immobilized ligands on the surface could be observed by significant changes in the RI near the gold surface. The home-made Kretschmann flow-cell (40 $\mu$L volume) was used for this purpose. The gold substrate (45 nm) was functionalized with polyclonal rabbit anti-*E. coli* antibodies using the amine coupling method, as described before^52–54^. Figure 6a shows the *in-situ* measurement for different steps of antibody immobilization on the gold surface. For *in-situ* measurements, the angle of incoming and reflecting arms in the PMSE setup were set to $75^{\circ }$. The flow speed of the solutions was set to 6 $\mu$L/min. The red and blue shifts of the SPR dip in the intensity spectra has been traced due to the change of RI of the target environment. This tracing was performed with 1 second time resolution (the highest time resolution is on the order of milliseconds). In order to estimate the limit of detection (LOD), six different concentrations (colony forming unit (CFU)/mL) of bacteria were prepared. The measurement was started from the lowest concentration ($10^2$ CFU/mL) to the highest concentration ($10^7$ CFU/mL). For each cycle, bacteria in solution were pumped for a period of 15 min and between each bacteria solution injection the surface was washed by PBS for 10 min. The *in-situ* spectra for each cycle (bacteria injection and washing process) are given in Fig. 6b. In the initial stages of the detection process, there is a noticeable increase, which can be caused by manually changing the pipe or using liquids with different temperatures (PBS and bacteria from incubators). The results of this experiment indicate that this biosensor is able to detect a minimum concentration of $10^2$ CFU/mL, which is the LOD for the developed PMSE. Arya et al. detected *E. coli* bacteria using SPR-based angular modulation. In their research, T4-bacteriophage was used as a linker to the gold surface and the detection limit of $10^{2}$ CFU/mL was reported^56^. The bacteriophages were also used by Tawil et al. for *E. coli * detection using the same method with $10^{3}$ CFU/mL the as the LOD^57^. Generally, the antibodies are used with SPR technique for *E. coli* detection and their LOD is in range of $10^{2}$ CFU/mL - $10^{4}$ CFU/mL^23–25^. These detection methods are limited to angular modulation-based measurements by using a single wavelength light source, while in this study a comparable LOD was reported for the wavelength modulation method. After washing the gold surface with PBS in the final step, an optical image was taken from the surface. Figure 6c, shows the single bacteria cells attached to the gold surface, and approximately 4.1${\%}$ of the surface is covered with bacteria. The changes in the position of the resonance wavelength ($\lambda _{SPR}$) corresponding to different concentrations of the bacteria was calculated and the quadratic fit is shown in Fig. 6d. A high correlation ($\hbox {R}^2$ = 0.98) between the position of the resonance wavelength and known bacteria concentration was observed. As a control experiment, a pure gold sample was tested, and the results are presented in the supplementary material. The PMSE-based SPR biosensor examined in this study was found to provide good performance while it is based on an uncomplicated optical setup in comparison to other selected methods^28,44,46–51^. The prominent feature of this work is that the sensor can be used for measurement in a wide range of wavelengths with high speed.

**Fig. 7:**
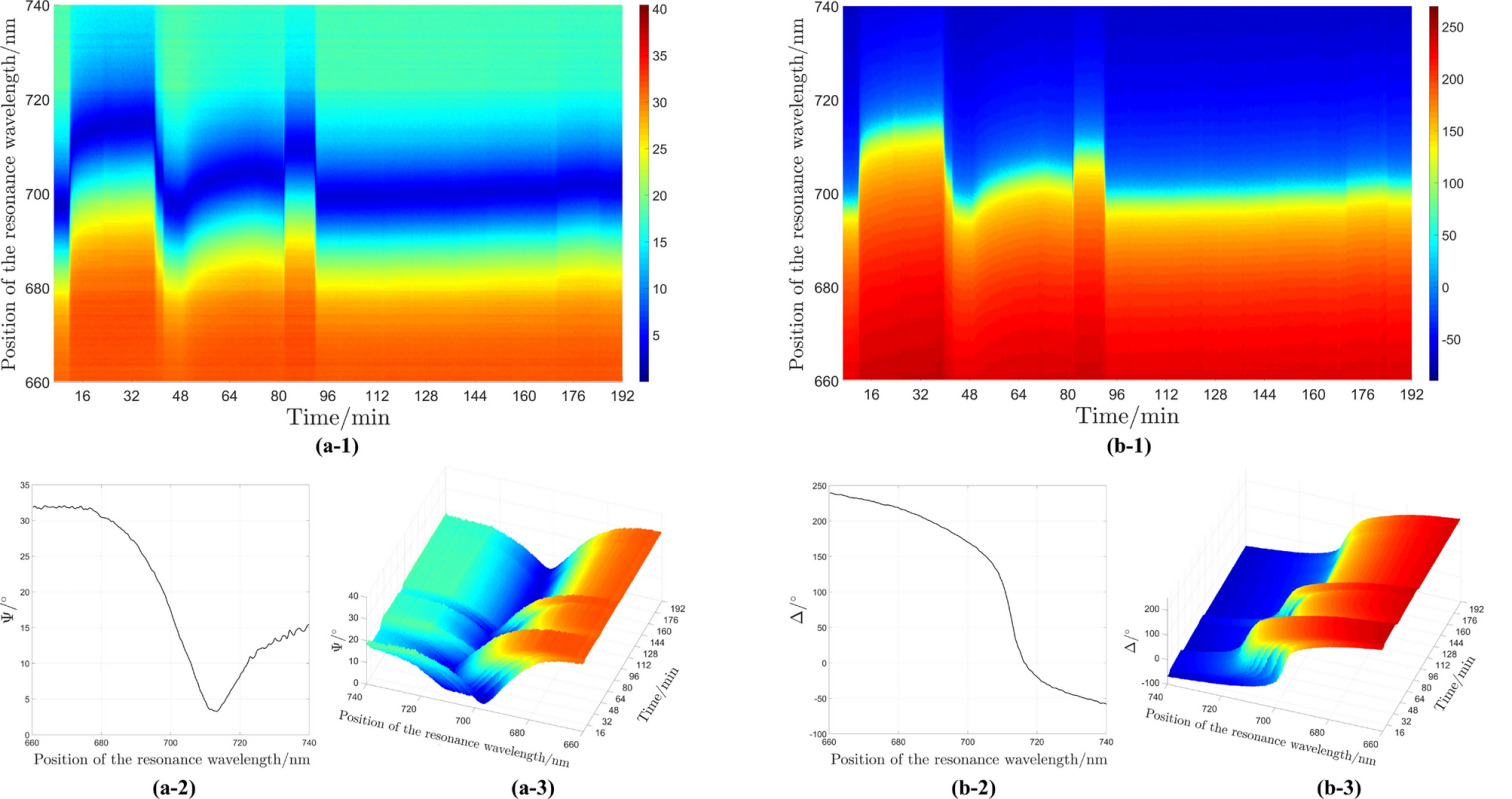
(**a-1**) and (**b-1**) Two-dimensional *in-situ* sensogram measurements including ligand immobilization and bacteria detection steps for $\Psi$ and $\Delta$, respectively. (**a-2**) and (**b-2**) Cross sections of $\Psi$ and $\Delta$ spectra, respectively. (**a-3**) and (**b-3**) Three-dimensional *in-situ* sensogram measurements for $\Psi$ and $\Delta$, respectively.

Using the developed PMSE optical setup in this study, *in-situ* SPR-based intensity and phase measurements were achieved based on the position of the resonance wavelength. In this configuration, the compensator has been taken into account and both polarizer and analyzer were set at $45 ^{\circ }$. The use of a light source with a wide spectrum in the optical setup makes it possible to evaluate the ellipsometric angles at different wavelengths. Figure 7 shows two- and three- dimensional graphs measured for the ellipsometric angles ($\Psi$ and $\Delta$). Results are shown for the entire process, including the immobilization of ligands and the detection of bacteria. Through the use of chemicals with different refractive indices, the immobilization of ligands can be visually distinguished from the detection of bacteria in Fig. 7a-1 and b-1. For $\Psi$, as the color of the spectrum shifts from orange to deep blue, the amount of reflected light decreases, and this means that the deep blue color in Fig. 7a-1 shows the location close to the plasmonic resonance. Additionally, in the $\Delta$ spectrum, the shift from orange to green indicates the area where a SPR occurred due to the shift in spectra. For better visualization of the diagrams, three-dimensional and cross sectional diagrams are also added. These measurements have a resolution of 100 milliseconds, which shows the capability of the optical setup for high resolution *in-situ* measurements of chemical and biological changes.

## Conclusions

The need to control the environmental surroundings for protection from chemical and biological contamination, makes it necessary to identify and control potential threats. Here, a developed PMSE was utilized for phase sensitive SPR measurements to detect *E. coli* K12, for the first time. The ellipsometric angles were measured using an electro-optic phase modulator which sets the phase shift with millisecond time resolution. This method also provides high time resolution information on both phase and intensity modulations over a wide range of wavelengths. The optimal thickness of the gold layer and the AOI were found at 45 nm and $75 ^{\circ }$, respectively. The PMSE has the capability of *in-situ* measurement of $\Psi$ and $\Delta$ spectra in the wavelength range from 350 nm - 850 nm. The sensitivity of the sensor shows linear behavior in the region of 1.3327 RIU - 1.3440 RIU with a maximum sensitivity of 3,858 nm$\cdot \hbox {RIU}^{-1}$ and a resolution of $10^{-5}$ RIU. The functionalized gold film with anti-*E. coli* antibody was tested with a series of bacteria and the LOD is $10^2$ CFU/mL. Further development of the biosensor performance will address applications for sorting and detecting complex biological targets and developing an optical model of the system to optimize its functionality.

## Supplementary Information

Supplementary Information.

## Data Availability

The datasets used and/or analyzed during the current study available from the corresponding author on reasonable request.
