# Supplementary material for: Polarization modulated spectroscopic ellipsometry-based surface plasmon resonance biosensor for E. coli K12 detection
Source: Sci Rep. 2024 Nov 7;14:27046. doi: 10.1038/s41598-024-78535-8 (PMC11544225; doi:10.1038/s41598-024-78535-8)
Supplement: Supplementary file 1 — Supplementary Information. [file 41598_2024_78535_MOESM1_ESM.pdf]

Supplemental material

Polarization modulated spectroscopic ellipsometry-based surface plasmon resonance biosensor for E. coli K12 detection

Soraya Zangenehzadeh, Emil Agocs, Fenja Schröder, Nassima Amroun, Rebekka Biedendieck, Dieter Jahn, Axel Günther, Lei Zheng, Bernhard Roth, Hans-Hermann Johannes, and Wolfgang Kowalsky

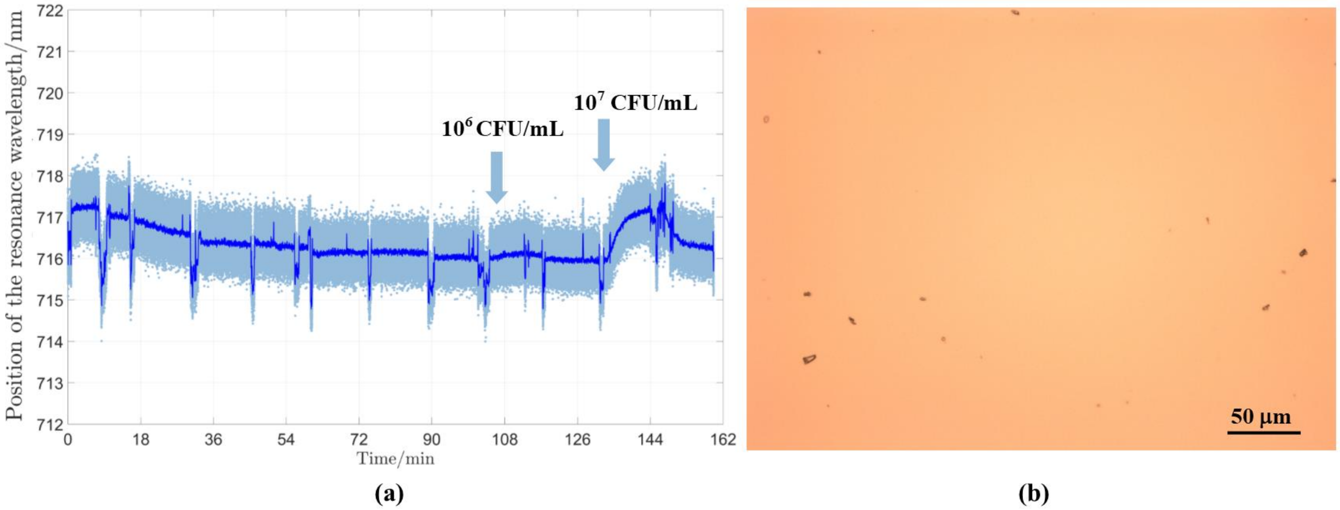

Figure 1. (**a**) Sensogram of bacteria injections through the surface from lowest to highest concentrations. Between each injection, PBS was used to wash the surface. (**b**) Optical image of the gold surface after washing with PBS.

As a control measurement, pure gold film samples without anti-*E. coli* antibody were tested. Figure 1 (a) shows the *in-situ* measurement for six different concentrations of bacteria. The measurement was started with PBS for 10 minutes followed by the lowest concentration of *E. coli* (10^2 CFU/mL) for 15 minutes. This cycle was repeated for each concentration, and finally, the surface was washed with PBS. As can be seen in Fig. 1 (a), only for the two highest concentrations the shift in the signal is clear enough to be recognized. The beginning of the shifts is identified with blue arrows for 10^6 and 10^7 CFU/mL, respectively. Figure 1 (b) shows the optical image of the gold surface. The control measurement demonstrates that only for high-concentration bacteria solutions, a signal can be observed. In this measurement, no bacteria are attached to the sensor surface after washing, and this shift in the signal originates from the changes in the refractive index of the environment close to the gold surface.
